# The Prevalence and Characteristics of Metabolic Syndrome in Patients with Vertigo

**DOI:** 10.1371/journal.pone.0080176

**Published:** 2013-12-03

**Authors:** Toshiaki Yamanaka, Takehiko Fukuda, Shiho Shirota, Yachiyo Sawai, Takayuki Murai, Nobuya Fujita, Hiroshi Hosoi

**Affiliations:** 1 Department of Otolaryngology-Head and Neck Surgery, Nara Medical University School of Medicine, Kashihara, Nara, Japan; 2 Department of Otorhinolaryngology, Nara Prefectural Hospital, Nara, Japan; Baylor College of Medicine, United States of America

## Abstract

**Objectives/Hypothesis:**

Metabolic syndrome (MetS) is a condition that increases the risk of coronary artery disease and cerebral infarction. We determined the prevalence of MetS in vertigo patients and clinically investigated the association between MetS and vertigo.

**Study Design:**

Case-control study

**Methods:**

The subjects were 333 patients, including 107 males and 226 females, who presented with vertigo as a primary symptom. MetS was diagnosed according to the International Diabetes Federation definition, which is based on waist circumference, blood serum levels, and blood pressure.

**Results:**

MetS was detected in 53 (15.9%) of 333 vertigo patients, including 24 males (22.4%) and 29 females (12.8%); i.e., the frequency of MetS was significantly higher among the male patients than the female patients. The overall prevalence of MetS (15.9%) among vertigo patients did not differ from that observed among general adults in previous Japanese surveillance studies; however, MetS was significantly more common among the vertigo patients in males than general adult males. The prevalence of MetS was also examined in five types of vertigo, Concomitant MetS was noted in many males with vertebrobasilar insufficiency (VBI) and isolated vertigo of unknown etiology.

**Conclusion:**

It was suggested that MetS is involved in the development of vertigo in males. MetS might be a risk factor for vascular vertigo such as VBI in males. The high frequency of MetS among males with vertigo of unknown etiology suggested that the pathogenesis of metabolic syndrome is involved in this type of isolated vertigo.

## Introduction

Lifestyle-related diseases, e.g., visceral obesity, high blood pressure (HBP), diabetes mellitus, and dyslipidemia (DLP), are considered to be independent risk factors for arteriosclerotic disease. It has recently been proposed that the accumulation of such risk factors synergistically increases the risk of cardiovascular disease and myocardial or cerebral infarction, even though the pathological levels of individual risk factors are mild, and this concept is referred to as metabolic syndrome (MetS) [1] [2]. Since the World Health Organization (WHO) initially published a definition and diagnostic criteria for MetS in 1999 [3], various definitions of MetS have been approved by international organizations or clinical expert study groups [4] [5] [6].

In addition, individual conditions such as HBP, diabetes, and DLP have been suggested to be associated with the prevalence of vertigo [7] [8] [9] [10], however, it is unclear whether MetS is associated with the onset of vertigo. In this study, we determined the prevalence of MetS in vertigo patients to investigate whether MetS involves in vertigo.

## Materials and Methods

This study was approved by the clinical research ethics board of Nara Medical University Hospital (Approval No. 620). All patients had received written instructions explaining this study, and they provided their verbal informed consent to participate in this study. The examinations involving blood tests, waist circumference measurements, blood pressure measurements, neurotological examinations and imaging studies for the present study were performed as routine primary tests for vertigo patients. The clinical research ethics board of our institute also approved verbal informed consent, because all the examinations involved in this study are conventional tests regularly performed for the patients with vertigo and do not give disadvantage to patients.

The subjects were 333 vertigo patients, including 107 males (age range: 21 to 84 years, mean: 59.8 years) and 226 females (age range: 21 to 87 years, mean: 58.0 years).

We subjected the patients to waist circumference measurements, blood tests, blood pressure measurements, and routine vestibular tests (neurotological tests and imaging studies) for vertigo patients. Waist circumference was measured in the horizontal plane, midway between the inferior margin of the ribs and the superior border of the iliac crest. Blood was sampled from patients who had fasted for at least four hours and used to measure their serum total cholesterol, high-density lipoprotein (HDL), cholesterol, triglyceride (TG), and glucose levels. Blood pressure was measured three times in the sitting position after at least 10 min of rest. Systolic and diastolic blood pressure values were determined as the mean of the second and third measurements, which were recorded at least 1 min apart using an automated sphygmomanometer (BP-103iII, Omron Colin, Tokyo, Japan).

In this study, MetS was diagnosed according to the International Diabetes Federation (IDF) MetS diagnostic criteria [4], which include waist circumference criteria for Asian people. According to this definition, the waist circumference cut-off values for Asians are 90 cm for males and 80 cm for females. In addition to central obesity as determined by this definition, a diagnosis of MetS also requires any two of the following four factors: (1) increased TG levels; i.e., serum TG levels ≥150 mg/dl and/or the current use of cholesterol-lowering medication, (2) reduced HDL-cholesterol levels; i.e., serum HDL levels of ≤40 mg/dl for men or ≤50 mg/dl for women, (3) increased blood pressure; i.e., systolic blood pressure (SBP)/diastolic blood pressure (DBP) values of ≥130/85 mmHg and/or the current use of antihypertensive medication, and (4) increased fasting plasma glucose levels; i.e., fasting blood glucose levels of ≥100 mg/dl and/or the current use of insulin or oral medication for diabetes (Table 1).

**Table 1:** Diagnostic criteria of the metabolic syndrome used in this study.

(1)Waist circumference	≳90 cm in men and ≳80 cm in women
(2)Raised TG level	serum TG levels ≳150 mg/dl and/or current use of cholesterol-lowering medication
(3)Reduced HDL cholesterol	serum HDL levels of ≤40 mg/dl in men and ≤50 mg/dl in women
(4)Raised blood pressure	SBP/DBP≳130/85 mmHg and/or current use of antihypertensive medication
(5)Raised fasting plasma glucose	fasting blood glucose ≳100 mg/dl and/or current use of insulin or oral medication for diabetes

TG, triglyceride; HDL, high-density lipoprotein; SBP, systolic blood pressure;

DBP, diastolic blood pressure.

The prevalence of MetS in vertigo patients was investigated according to gender, age, and disease. We compared our data with the surveillance data for Japanese adults obtained by the DECODA study group [11], because this study is the only report which describes the number of people with MetS according to age group and gender. The Chi-square test or Fisher's exact test was employed for statistical analyses in this study.

## Results

MetS was detected in 53 (15.9%) of the 333 vertigo patients examined in this study, including 24 of 107 males (22.4%) and 29 of 226 females (12.8%); i.e., the prevalence of MetS was almost two-fold higher (P<0.05) among the male patients than among the female patients.

**Figure 1:**
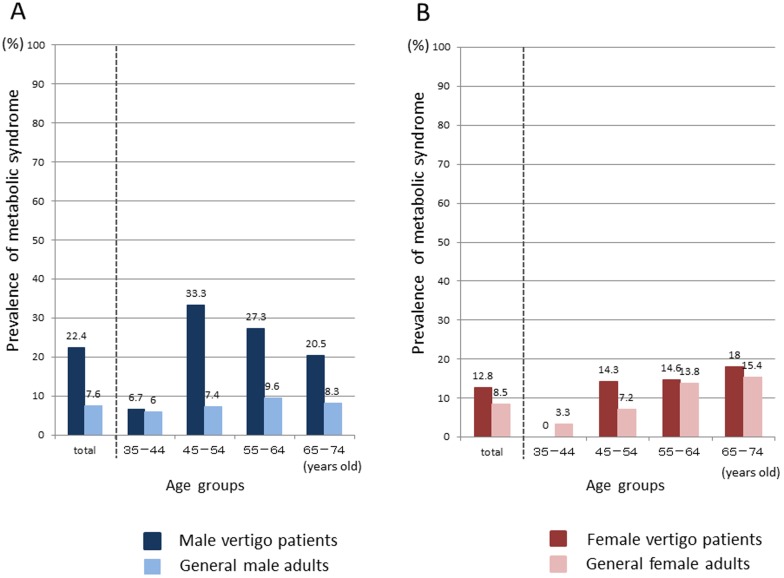
Prevalence of metabolic syndrome by age and gender in vertigo patients and general adults. This figure shows prevalence of metabolic syndrome (MetS) in male (A) and female (B) vertigo patients and in general adults according to age group and gender. The data of general adults were obtained by a Japanese surveillance study (the DECODA study group). Numerical values depict the prevalence rate (%) of MetS.

Figure 1 shows the prevalence of MetS among the male and female vertigo patients according to age group. For comparison, surveillance data for Japanese adults obtained by the DECODA study group [11] is also shown. The male vertigo patients exhibited an approximately three-fold higher (P<0.01) prevalence (22.4%) of MetS than the adult males (7.6%) in the DECODA study (Fig. 1A) (Table 2). A higher prevalence of MetS was observed among the male vertigo patients in every age group. It was highest in the 45–54-year-old age group (Fig. 1A). As for females, the prevalence (12.8%) of MetS was slightly, but not significantly, higher among the vertigo patients than (8.5%) among the general adult females (Fig. 1A) (Table 2). However, differences in the prevalence of MetS were seen between the age groups in the adult females; i.e., it was lower among the vertigo patients than among general adults in the 35–44 years old age group, while it was slightly higher among the vertigo patients in the 45–54-year- old age group, and it was about the same in the 55 years or older age group (Fig. 1B).

**Table 2:** Prevalence of metabolic syndrome in vertigo patients and general adults.

		Metabolic syndrome		*P* value
		present	absent	
male	Vertigo patients	24(22.4)	83	*p*<0.01
	General adults	58(7.6)	708	
female	Vertigo patients	29(12.8)	97	NS
	General adults	91(8.5)	977	

The data for general adults were obtained by a Japanese surveillance study (the DECODA study group).

Numerical values depict the number of patients.

Numerical values in parenthesis depict the proportion of MetS (%).

NS, not significant.

The prevalence of MetS was also examined in five types of vertigo, each of which was displayed by at least twenty of the patients examined in this study (Table 3). MetS was detected in 10 (16.4%) of 61 patients with Meniere's disease (MD), 7 (14%) of 50 patients with benign paroxysmal positional vertigo (BPPV), 7 (25%) of 28 patients with vertebrobasilar insufficiency (VBI), 4 (10.5%) of 38 patients with orthostatic disorder (OD), and 12 (18.5%) of 65 patients with isolated vertigo of unknown etiology. The VBI and OD patients displayed the highest (25%) and lowest (10.5%) MetS frequencies, respectively.

**Table 3:** Prevalence of the metabolic syndrome by gender in each disease type of vertigo.

	number of case	total	male	female
Meniere's disease	61	10/61 (16.4%)	5/22 (22.7%)	5/39 (12.8%)
benign paroxysmal positional vertigo	50	7/50 (14%)	1/11 (9.1%)	6/39 (15.4%)
vertebro-basilar insufficiency	28	7/28 (25%)	5/13 (38.5%)	2/15 (13.3%)
orthostatic disorder	38	4/38 (10.5%)	1/7 (14.3%)	3/31 (9.7%)
etiology-unknown vertigo	65	12/65 (18.5%)	7/20 (35%)*	5/45 (11.1%)

* : P<0.05 vs the prevalence of MetS in females.

The incidence of MetS was investigated according to gender in each type of vertigo (Table 3). Among the VBI patients, who displayed the highest prevalence of MetS, MetS was detected in 5 (38.5%) of 13 males and 2 (13.3%) of 15 females; i.e., MetS was markedly more common in males with VBI. As for the other types of vertigo, the prevalence rates of MetS in males and females with MD were 22.7 (5/22) and 12.8% (5/39), respectively, those for BPPV were 9.1 (1/11) and 15.4% (6/39), respectively, those for OD were 14.3 (1/7) and 9.7% (3/31), respectively, and those for vertigo of unknown etiology were 35 (7/20) and 11.1% (5/45), respectively, showing that the MetS was more common in males than females in all types of vertigo except BPPV. Significant higher proportion (P<0.05) of MetS was detected in males with isolated vertigo of unknown etiology than females (Table 3).

## Discussion

In 1988, Reaven described combinations of metabolic disturbances as the term ‘Syndrome X’ and firmly established the clinical importance of the syndrome [12]. In 1989, Kaplan added obesity to the syndrome and renamed it ‘The Deadly Quartet’ [13], and others then coined the term ‘The Insulin Resistance Syndrome’ [14]. The term ‘metabolic syndrome’ is now the most widely accepted description of this cluster of metabolically related cardiovascular risk factors. MetS criteria have been proposed by different organizations; the European Group for the Study of Insulin Resistance [6], the National Cholesterol Education Program-Third Adult Treatment Panel [5], and the IDF [4], since the WHO initially published a definition and diagnostic criteria for MetS in 1999 [3]. However, the waist circumference criteria for MetS remain unclear because the relationships between waist circumference and the risk of cardiovascular disease or diabetes exhibit racial differences, and there is a lack of clarity about their role in clinical practice. In an attempt to resolve this issue and establish universal criteria for MetS, a joint interim statement by the American Heart Association/National Heart, Lung, and Blood Institute; the IDF; the World Heart Federation, the International Atherosclerosis Society, and the International Association for the Study of Obesity in 2009 [15] included an agreed definition of MetS, which advocated national or regional cut-off points for waist circumference [15] [16]. We used the Japanese criteria proposed by the Japanese Society of International Medicine (85 cm for men, 90 cm for women) [17] to define MetS in vertigo patients in our previous report in Japanese [18], however, this interim statement recommends that the IDF waist circumference criteria should be applied to Japanese people. Thus, we used these IDF criteria (90 cm for men, 80 cm for women) to diagnose MetS in this study [15] [16].

In the current study, we examined 333 patients with vertigo, 15.9% (53 patients) of whom exhibited MetS, and this proportion was not markedly different from those (8.1% [11], 16.6% [19], and 15.4% [20]) reported for Japanese adults in previous surveys. These surveillance reports also described that MetS was less common in males than in females although differences in the prevalence rate were seen among these studies. However, our study found that MetS was present in 22.4% of male vertigo patients, but only 12.8% of female vertigo patients; i.e., the frequency of MetS was significantly higher in male vertigo patients. Comparing the MetS prevalence data obtained in our study with the data obtained by a previous Japanese surveillance study, according to age group and gender, revealed that the prevalence rate of MetS in males with vertigo is significantly higher than that in the general adult males and MetS is more common in males with vertigo in all age groups. These results suggest that MetS is involved in the development of vertigo in males.

MetS is considered to damage the vascular endothelium, and hence, promote inflammation and blood coagulation in the vasculature, leading to the development of arteriosclerosis [21]. Thus, vascular disorders involving the vestibular nervous system might be involved in the onset of vertigo in patients with concomitant MetS. Actually, the prevalence of MetS was highest (25%) among the patients with VBI. MetS might induce vascular disorders involving the vertebrobasilar arterial system and initiate the pathology of VBI. Since the risk of cerebrovascular disorders, such as stroke and cerebral infarction, generally increases in patients with MetS [1] [2] [22], VBI with MetS might require careful follow-up and treatment [23] aimed at preventing the progression of cerebrovascular disorders.

In this study, isolated vertigo of unknown etiology displayed a much higher frequency of MetS (35%) than is seen in general male adults. Isolated vertigo is difficult to diagnose; however, our findings suggest that some patients with MetS who present with isolated episodes of vertigo might be suffering from vascular dysfunction involving the vestibular nervous system. Based on this, we propose MetS-associated vertigo as a new disease type of vertigo which could be included in some male patients with isolated vertigo of unknown etiology.

This study suggests that screening for MetS might be important for preventing the onset of vertigo caused by underlying vascular disorders. However, it is unclear whether MetS is actually a risk factor for vertigo because there have not been any epidemiological surveys of the prevalence of vertigo in people with MetS. A long-term prospective epidemiological survey of vertigo in the general public is required.

## Conclusion

The results of our study suggest that MetS might be closely associated with the pathogenesis of vertigo in some cases involving vascular disorders affecting the vestibular nervous region, and that vascular dysfunction might be an important shared mechanism underlying isolated episodes of vertigo. However, data were obtained from a single institute in this study; therefore, the possibility that our results are affected by regional differences cannot be excluded. Further studies are required to determine whether our results are consistent with the general population.
